# Dietary Habits and Atopic Dermatitis Significantly Influence the Fecal Microbiome

**DOI:** 10.1111/all.70459

**Published:** 2026-07-28

**Authors:** Danielle Fehr, Nicole Flack, Gloria Masenga, Nelson Mosha, Nick Li, Anna White, George Semango, Meike Distler, Claudia Lang, Felix Grimm, Michael Scharl, Daudi Mavura, John Elisante Masenga, Peter Schmid‐Grendelmeier, Marie‐Charlotte Brüggen

**Affiliations:** ^1^ Allergy Unit, Department of Dermatology University Hospital Zurich Zurich Switzerland; ^2^ Faculty of Medicine University of Zurich Zurich Switzerland; ^3^ Christine Kühne‐Center for Allergy Research and Education Davos Switzerland; ^4^ Department of Internal Medicine Kantonsspital Münsterlingen Münsterlingen Switzerland; ^5^ Genevia Technologies Oy Tampere Finland; ^6^ Regional Dermatology Training Center Kilimanjaro Christian Medical Center Moshi Tanzania; ^7^ School of Medicine Kilimanjaro Christian Medical Centre University Moshi Tanzania; ^8^ Department of Microbiology, Parasitology and Biotechnology College of Veterinary Medicine and Biomedical Sciences, Sokoine University of Agriculture Morogoro Tanzania; ^9^ Department of Cardiology Hochgebirgsklinik Davos Davos Switzerland; ^10^ Institute of Parasitology, University of Zurich Zurich Switzerland; ^11^ Department of Gastroenterology and Hepatology University Hospital Zurich Zurich Switzerland

**Keywords:** atopic dermatitis, gut, metagenomics, microbiome

## Abstract

**Background:**

Atopic dermatitis (AD) has been associated with microbial gut dysbiosis in children. Data in adults is scarce.

**Objective:**

We sought to explore the fecal microbiome composition in AD patients versus healthy controls (HC) and investigate the impact of environmental aspects such as the geographical location and dietary habits.

**Methods:**

In this case control study, a total of 140 fecal samples from 69 ad patients and 71 HC from Tanzania (TZ) and Switzerland (CH) were analyzed. Illumina shotgun metagenomics sequencing was performed followed by taxonomic profiling, calculation of alpha/beta diversity, and permutational multivariate analysis of variance. Differentially abundant species and genera between AD and HC were evaluated.

**Results:**

Alpha diversity (Shannon Index) did not significantly differ between AD and HC. The presence of AD was identified as a significant source of fecal microbiome variation (Bray–Curtis beta diversity). On the species level, *Faecalibacterium taiwanense* was more abundant in AD, and *Vescimonas sp000435555* in HC. On the genus level, CAG‐302 (NCBI: Clostridium) was more abundant in HC. Geographical location was associated with distinct dietary habits. *Prevotella* was significantly more abundant in TZ, whereas *Alistipes* and *Bacteroides* were significantly more abundant in CH.

**Conclusions:**

The presence of AD significantly impacted the fecal microbiome variation and was associated with a particular microbial composition. The impact of geographical location on the fecal microbiome may be related to nutritional differences. Our data support the rationale of a gut‐skin axis and pave the way towards therapeutic approaches modulating the microbiome.

## Graphical abstract

Atopic dermatitis, dietary habits, sex, and BMI significantly influenced the fecal microbiome composition. On the species level, *Faecalibacterium taiwanense* was more abundant in atopic dermatitis, and *Vescimonas sp000435555* was more abundant in the control population. On the genus level, CAG‐302 (NCBI: *Clostridium*) was more abundant in controls. The severity of atopic dermatitis as well as atopic comorbidities did not show a significant impact on gut microbiome variation. AD, atopic dermatitis; BMI, body mass index; IgE, immunoglobulin E; IgG, immunoglobulin G.

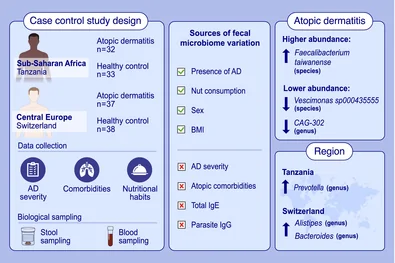

**Abbreviations**
AD: atopic dermatitis
ADAC: atopic dermatitis with atopic comorbidities
ADpure: atopic dermatitis without atopic comorbidities
BMI: body mass index
BSA: body surface area
CH: Switzerland
EASI: Eczema Area and Severity Index
ELISA: enzyme‐linked immunosorbent assay
FMT: fecal microbiota transplantation
GTDB: gene taxonomy database
IgE: immunoglobulin E
IgG: immunoglobulin G
NCBI: National Center for Biotechnology Information
oSCORAD: objective SCORAD
PPA: phosphopantothenate
PoB: positive or borderline
SCORAD: Scoring Atopic Dermatitis
SGB: species‐level genome bin
SS: sum of squares
Th2: T helper 2
TZ: Tanzania
vIGA‐AD: Validated Investigator Global Assessment scale for Atopic Dermatitis

## 1 Introduction

Atopic dermatitis (AD) is a common chronic inflammatory skin disease, which can severely affect the patients' quality of life [1]. An interplay of epidermal barrier dysfunction and type 2 immune response/immune dysregulation is a central process in AD pathogenesis [2, 3, 4].

Furthermore, changes in the skin microbiome were found in AD lesions and have been associated with disease severity and exacerbations [5, 6, 7]. The role of *Staphylococcus aureus*, but also other bacteria, has been extensively characterized [7, 8, 9, 10]. Increasing attention is attributed to the gut counterpart of the skin microbiome in AD based on the concept of the “gut‐skin axis”. A study in children showed an association between AD and an increased relative abundance of *Clostridium sensu stricto* in fecal samples in 3‐month‐old babies [11]. Other reports described changes in the gut microbiome in AD, such as a subspecies‐level enrichment of *Faecalibacterium prausnitzii* [12, 13]. Gut microbial changes were also described in other atopic diseases such as asthma or food allergies [14, 15]. The exact mechanisms involved in AD currently remain unclear; recent data suggested a bacterial dysbiosis promoting AD inflammation, partially via systemic effects of microbial metabolites [16, 17, 18, 19]. Some bacterial metabolites have immunomodulatory effects: butyrate can induce the differentiation and expansion of regulatory T cells by epigenetically modulating gene expression [20]. In AD, regulatory T cells are involved in attenuating chronic skin inflammation [19, 21, 22]. A study in children with AD showed a lower likelihood of high butyrate levels in children with AD [23]. Other gut microbes' roles in AD are being investigated. This also applies to their potential therapeutic exploitation: Probiotics/butyrate supplements and fecal microbiota transplantation (FMT) from healthy donors to AD patients have been suggested [24, 25, 26, 27, 28].

The fecal microbiome is known to be influenced by multiple factors ranging from age, infections, weight/obesity to nutrition and medication intake [14, 29, 30, 31, 32, 33]. Many of these factors are environment‐associated, such as nutrition or infections. Regarding the latter, helminth infections for instance have been shown to alter the gut microbiome [32].

AD is heavily affected by various environmental factors such as irritants/pollutants or allergen exposure, notably via their negative impact on the skin barrier and type 2 inflammation [34, 35, 36, 37]. A better knowledge of whether and how these factors influence a potential gut microbial dysbiosis in AD is warranted to understand the impact on cutaneous and systemic inflammation. This may ultimately help us develop new therapeutic approaches.

Our main question was whether and how gut dysbiosis is impacted by the presence of AD and different environmental factors. More specifically, we hypothesized that there are differences in the gut microbiome of AD patients and healthy controls (HC) from Switzerland (CH, Europe) vs. Tanzania (TZ, sub‐Saharan Africa). This may mainly be attributable to differences in dietary intake (i.e., nutrition) in these different geographical areas. Differences in the gut microbiome may in turn impact the polarization/type of cutaneous and systemic inflammation in AD.

## 2 Methods

### 2.1 Study Design

In this case control study, a total of 140 participants were included. These consisted of AD patients residing in CH (*n* = 37) and TZ (*n* = 32) as well as HC participants (CH *n* = 38; TZ *n* = 33). Participants were included at one of the study centers (Zurich, CH and Moshi, TZ) between April 2022 and February 2024. AD patients and HC were included in the study if they were 18 years or older. AD patients were suffering from active AD at the time of inclusion. Detailed eligibility criteria can be found in the Supporting Information.

The study was conducted according to the Helsinki Declaration and ethical guidelines at the respective institutions. It was approved by the necessary local ethics committees (ClinicalTrials.gov Identifier: NCT05363904, CH: BASEC 2021‐01869, TZ: NIMR Nr. 3708). All participants gave their written informed consent.

### 2.2 Clinical Data Collection

Data on lifestyle habits (such as smoking) were collected in a standardized way during a physician‐patient interview. AD severity was assessed by a trained physician using EASI (*Eczema Area and Severity Index*), *SCORAD* (*Scoring Atopic Dermatitis* [38]), and vIGA‐AD (*Validated Investigator Global Assessment scale for Atopic Dermatitis*). Dietary and lifestyle habits were self‐reported by the patients and assessed in a standardized questionnaire. Mean dietary scores were calculated across specific food items for 12 major categories (dairy, meat, crustaceans and mollusks, fish, vegetables, cereals and pseudocereals, seeds, legumes, nuts, fruit, fermentation ingredients (yeast and malt), meal frequency), then the composite scores were centered and scaled across the cohort. Patients were stratified into AD with and AD without atopic comorbidities (ADAC and pure AD) according to the reported presence of allergic rhinitis, allergic rhinoconjunctivitis, asthma, and/or food allergies.

### 2.3 Serum Sample Collection

Blood sampling was performed in all patients who gave their written informed consent. After a clotting time of 1–4 h at room temperature, centrifugation at 1300× *g* for 10 min was performed, and the serum supernatant collected. The serum was stored at −80°C until further use.

### 2.4 Fecal Sample Collection

A stool sampling kit (OMNIgene GUT, OMR‐200, DNA Genotek, Ottawa, Canada) was handed out to the participants upon the study visits. Participants were taught to perform the sampling according to the manufacturer's instructions and provide the stool sample to the laboratory. Stool samples were stored at −80°C until further use.

### 2.5 DNA Isolation and Sequencing

Nucleic acids were extracted using the Tiangen Magnetic Soil and Stool Kit (Beijing, China) according to the manufacturer's instructions. Shotgun metagenomics sequencing was performed using an Illumina NovaSeq X Plus Series platform using a PE150 approach (150 bp paired‐end reads) to generate > 12 Gb of raw data per sample (Novogene GmbH, Munich, Germany). Therefore, the genomic DNA was randomly cut into fragments which were then end repaired, A‐tailed and ligated with an Illumina adapter. Fragments with adapters were amplified with PCR, followed by size selection and purification. Quantification was checked with Qubit and real‐time PCR and size distribution with bioanalyzer before pooling and sequencing of quantified libraries on Illumina platforms. Raw sequencing reads are publicly available through the European Nucleotide Archive (study accession: PRJEB101120).

### 2.6 Parasite Screening

A serological screening for various helminth immunoglobulin G (IgG) antibodies was performed using enzyme‐linked immunosorbent assays (ELISA). Serum was screened for IgG to *Echinococcus granulosus*, *Echinococcus multilocularis*, *Fasciola*, *Filariae*, *Schistosoma*, *Strongyloides*, *Toxocara*, *Trichinella*, and *Taenia solium*.

### 2.7 Statistical Analysis

Quality check of FASTQ files were performed using fastp [39] v0.23.4 and MultiQC [40] v1.14. MetaPhlAn4 [41] v4.1.1 was used to calculate as genus‐ and species‐level relative abundances with the ChocoPhlAn marker database “mpa_vJun23_CHOCOPhlAnSGB_202403”. Shannon indices were calculated on a species level and compared with pairwise Wilcoxon rank sum tests. The R package vegan [42] v2.6‐4 was used to calculate Bray–Curtis distances from species‐level relative abundance values, thereby excluding taxa present in < 10% of samples or with < 0.01% mean relative abundance. Associations between Bray–Curtis distances and AD status (presence, severity), atopic comorbidities, dietary intake, and further variables (e.g., total immunoglobulin E (IgE), parasites) were tested using PERMANOVA (vegan::adonis2 [42]) with type III sum of squares (SS; by = “margin”). All models included age, sex, and BMI as covariates, and predictors with *p* < 0.05 were considered significant. Differences in standardized diet scores between groups were tested using Mann–Whitney *U* tests; *p*‐values were adjusted for multiple testing using the Benjamini‐Hochberg procedure. The abundance of genes associated with microbial metabolic pathways (MetaCyc) was evaluated with HUMAnN [43] v3.9. Differentially abundant species, genera, and pathways between AD cases and controls were evaluated with MaAsLin2 [44] v1.12.0. Multiple tests were controlled with false discovery rate correction calculated by the Benjamini‐Hochberg method with *q*‐values < 0.1 (MaAsLin2 [44] default = 0.25) defined as significant. Affinity Designer 2 v2.6.5 was used to create the Graphical Abstract. Detailed information about the statistical methodology can be found in the Supplementary Methods (Online Repository) [45, 46, 47].

## 3 Results

### 3.1 Patient Characteristics

A total of 140 participants were included. These included 69 ad patients (CH: 37, TZ: 32) and 71 HC participants (CH: 38, TZ: 33). 41.7% (*n* = 58) were male, the median age was 31 years (range 18–84). The median SCORAD of AD patients was 31.6 (range 10.2–79.3). In total, 40.6% (*n* = 28) of AD patients reported a food allergy, 69.6% (*n* = 48) suffered from allergic rhinoconjunctivitis symptoms, and 27.5% (*n* = 19) reported allergic asthma. HC participants were non‐atopic by definition. Detailed patient characteristics can be found in Table 1.

**TABLE 1:** Patient characteristics of patients with atopic dermatitis and healthy control participants divided by region.

	Atopic dermatitis		Healthy controls	
	Switzerland (*n* = 37)	Tanzania (*n* = 32)	Switzerland (*n* = 38)	Tanzania (*n* = 33)
Sex				
Male	17 (45.9%)	11 (34.4%)	12 (31.6%)	18 (54.5%)
Female	20 (54.1%)	20 (62.5%)	26 (68.4%)	15 (45.5%)
Age				
Mean (SD)	30.9 (10.4)	44.6 (18.3)	32.9 (12.7)	38.2 (11.9)
Median [min, max]	28.0 [18.0, 61.0]	43.5 [19.0, 84.0]	28.0 [18.0, 61.0]	37.0 [19.0, 63.0]
BMI				
Mean (SD)	23.8 (3.33)	27.0 (5.60)	23.6 (3.99)	27.2 (5.46)
Median [min, max]	23.6 [18.1, 30.5]	25.2 [18.8, 40.4]	23.3 [17.3, 36.8]	25.7 [19.5, 39.6]
Allergic rhinoconjunctivitis				
No	9 (24.3%)	12 (37.5%)	38 (100%)	33 (100%)
Yes	28 (75.7%)	20 (62.5%)	0 (0%)	0 (0%)
Asthma				
No	26 (70.3%)	24 (75.0%)	38 (100%)	33 (100%)
Yes	11 (29.7%)	8 (25.0%)	0 (0%)	0 (0%)
Food allergy				
No	18 (48.6%)	23 (71.9%)	38 (100%)	33 (100%)
Yes	19 (51.4%)	9 (28.1%)	0 (0%)	0 (0%)
SCORAD				
Mean (SD)	29.8 (13.4)	39.6 (18.0)	NA (NA)	NA (NA)
Median [min, max]	27.2 [13.9, 75.6]	36.2 [10.2, 79.3]	NA [NA, NA]	NA [NA, NA]
EASI				
Mean (SD)	5.91 (6.41)	9.71 (10.7)	NA (NA)	NA (NA)
Median [min, max]	3.80 [0.200, 29.3]	4.50 [0.200, 42.0]	NA [NA, NA]	NA [NA, NA]
vIGA‐AD				
Mean (SD)	2.32 (0.852)	2.44 (0.840)	NA (NA)	NA (NA)
Median [min, max]	2.00 [1.00, 4.00]	2.00 [1.00, 4.00]	NA [NA, NA]	NA [NA, NA]
Total IgE				
Mean (SD)	1243 (1678)	1857 (1921)	150 (424)	558 (943)
Median [min, max]	364 [0, 5596]	1130 [0, 6886]	45 [0, 2386]	133 [0, 4029]

Non‐atopic systemic comorbidities are summarized in Table 2. Several country‐ and disease‐related differences were observed. Psychiatric disorders were significantly more frequently reported in patients with AD (*n* = 12, 17.4%), than in HC participants (*n* = 4, 5.6%, *p* = 0.03). In addition, psychiatric disorders were markedly more common in the Swiss than in the Tanzanian cohort (CH: *n* = 15, 20.0%, TZ: *n* = 1, 1.5%, *p* < 0.001). Hypertension was significantly more often reported in TZ AD patients (*n* = 11, 34.4%) than in CH AD patients (*n* = 1, 2.7%, *p* < 0.001). Gastrointestinal diseases were also reported more frequently in TZ AD patients (*n* = 4, 12.5%) than in CH AD patients (*n* = 2, 5.4%), although this difference did not reach statistical significance (*p* = 0.41). Likewise, no significant differences in gastrointestinal diseases were observed between centers overall (*p* = 0.08) or between AD patients and HC participants overall (*p* = 0.32). All reported gastrointestinal diseases were acid‐peptic disorders (e.g., gastritis, reflux). Neurological disorders were reported predominantly among CH AD patients (*n* = 4, 10.8%). Cardiovascular events (myocardial infarction, stroke), valvular heart disease, and renal or hepatic insufficiency were not reported in any group.

**TABLE 2:** Non‐atopic systemic comorbidities reported by participants, stratified by country (Switzerland, Tanzania) and group (atopic dermatitis, healthy controls).

	Atopic dermatitis		Healthy controls	
	Switzerland (*n* = 37)	Tanzania (*n* = 32)	Switzerland (*n* = 38)	Tanzania (*n* = 33)
Hypertension				
No	36 (97.3%)	21 (65.6%)	37 (97.4%)	32 (97.0%)
Yes	1 (2.7%)	11 (34.4%)	1 (2.6%)	1 (3.0%)
Hyperlipidemia				
No	36 (97.3%)	30 (93.8%)	37 (97.4%)	32 (97.0%)
Yes	1 (2.7%)	1 (3.1%)	1 (2.6%)	1 (3.0%)
Diabetes mellitus				
No	37 (100%)	30 (93.8%)	38 (100%)	32 (97.0%)
Yes	0 (0%)	2 (6.3%)	0 (0%)	1 (3.0%)
History of deep vein thrombosis				
No	37 (100%)	32 (100%)	37 (97.4%)	33 (100%)
Yes	0 (0%)	0 (0%)	1 (2.6%)	0 (0%)
History of pulmonary embolism				
No	36 (97.3%)	32 (100%)	38 (100%)	33 (100%)
Yes	1 (2.7%)	0 (0%)	0 (0%)	0 (0%)
Cardiac arrhythmia				
No	35 (94.6%)	31 (96.9%)	38 (100%)	33 (100%)
Yes	2 (5.4%)	1 (3.1%)	0 (0%)	0 (0%)
Gastrointestinal disease				
No	35 (94.6%)	28 (87.5%)	38 (100%)	30 (90.9%)
Yes	2 (5.4%)	4 (12.5%)	0 (0%)	3 (9.1%)
Neurological disorder				
No	33 (89.2%)	32 (100%)	37 (97.4%)	33 (100%)
Yes	4 (10.8%)	0 (0%)	1 (2.6%)	0 (0%)
Psychiatric disorder				
No	26 (70.3%)	31 (96.9%)	34 (89.5%)	33 (100%)
Yes	11 (29.7%)	1 (3.1%)	4 (10.5%)	0 (0%)
Other chronic or active disease				
No	30 (81.1%)	31 (96.9%)	33 (86.8%)	33 (100%)
Yes	7 (18.9%)	0 (0%)	5 (13.2%)	0 (0%)

*Note:* Comorbidities for which no events were recorded across all four groups (history of myocardial infarction, stroke, valvular heart disease, renal insufficiency, and hepatic insufficiency) are not displayed. Values are *n* (%).

### 3.2 Presence of AD as Significant Predictor of Microbiome Composition

First, we looked at the overall gut microbiome in our study population. Across all samples, a mean of 84.40% of sequences was successfully classified. Of the classified sequences, 99.74% were bacterial, 0.19% of eukaryotic origin, and 0.07% belonged to Archaea (Supporting Information Figure E1). The most abundant species included *Prevotella*, *Phocaeicola*, and *Bacteroides* species (Figure 1A,B).

**FIGURE 1:**
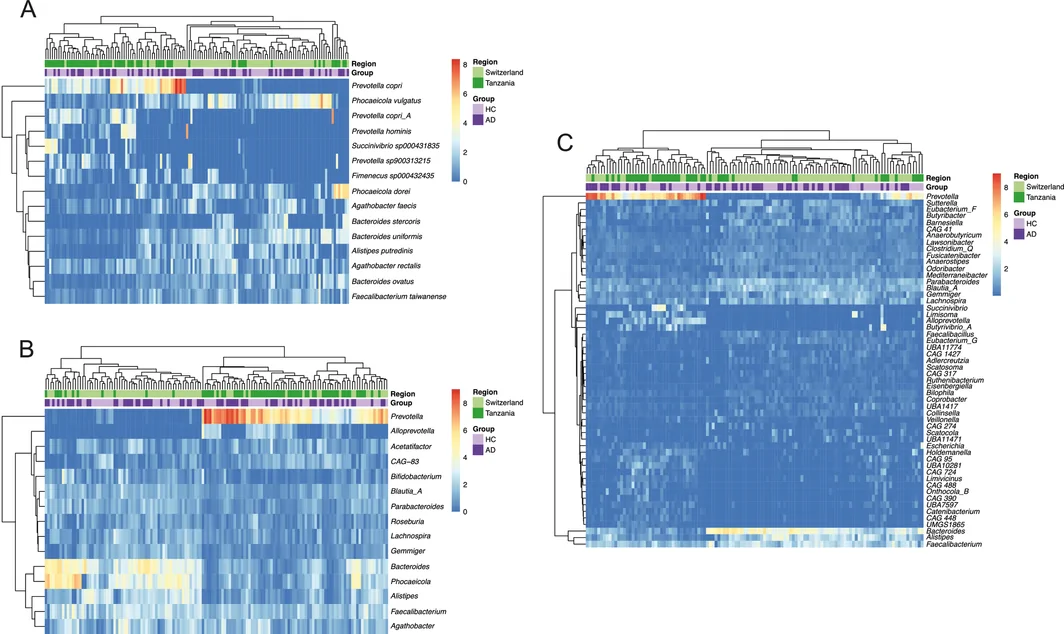
Heatmap of the top 15 most abundant (A) species and (B) genera by relative abundance. (C) Heatmap of the 54 significantly differentially abundant (*q* < 0.1) genera associated with region output by Maaslin2. The Maaslin2 model accounted for age, sex, and BMI; region also represented dietary variables in the model due to regional collinearity. The color bar indicates the square root transformed relative abundance value. AD, atopic dermatitis; HC, healthy control.

We then wanted to explore whether AD patients may exhibit differences in the gut microbiome compared to HC. The Shannon Index used for measuring alpha diversity in our study considers numbers of species (richness) and their relative abundances (evenness) in a sample [48] (Supporting Information Table E3). There was no significant difference in the alpha diversity of the microbiome depending on the presence vs. absence of AD (*p* = 0.402) (Figure 2A). Disease severity (EASI levels, vIGA‐AD) was also not associated with such differences (*p* = 0.87, *p* = 0.55; Figure 3).

**FIGURE 2:**
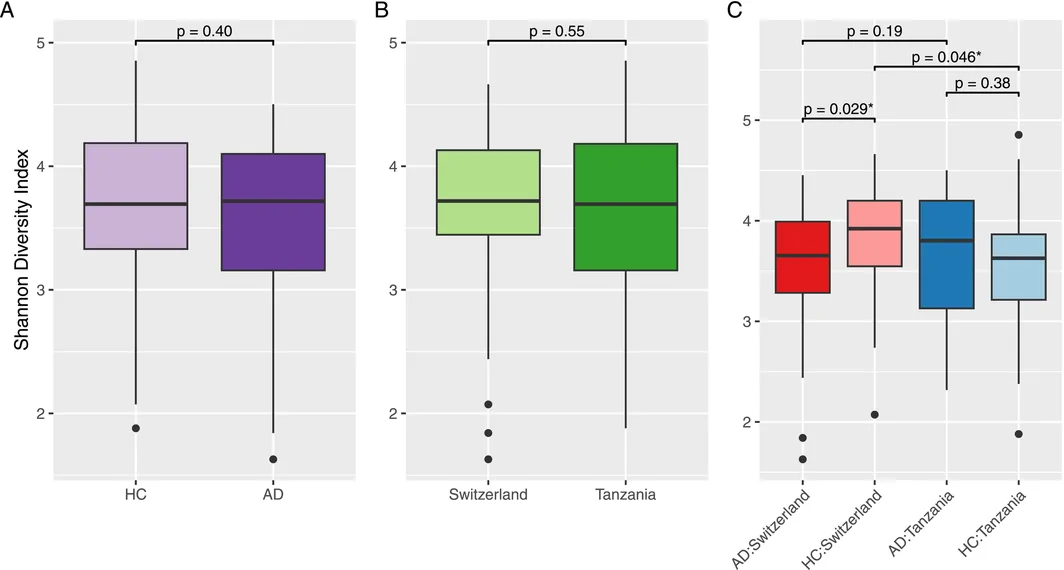
Differences in microbial alpha diversity (Shannon index) stratified by (A) AD presence, (B) region, and (C) combined AD × region categories. *p*‐Values were generated using two‐sided Wilcoxon rank‐sum tests. *p < 0.05. AD, atopic dermatitis; HC, healthy control.

**FIGURE 3:**
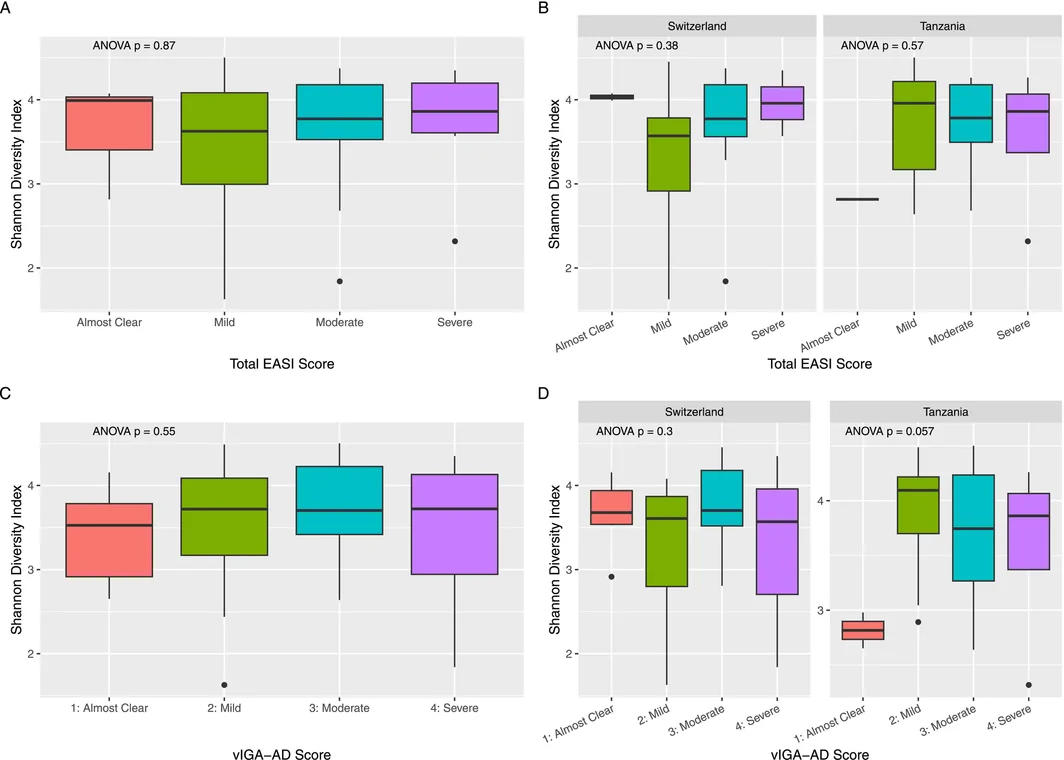
Boxplots of alpha diversity values (Shannon index) for AD patients by (A) EASI, (B) EASI split by region, (C) vIGA‐AD, and (D) vIGA‐AD split by region.

Species‐level Bray–Curtis dispersions (*n* = 139) were also similar between AD and HC (*p* = 0.40). AD presence did not reach statistical significance (*p* = 0.062) even when adjusting for age, BMI, and sex in a basic PERMANOVA model (type III SS, permutations stratified by region, Figure 4). BMI, however, was significantly associated with microbiome structure in TZ (*R* ^2^ = 3.0%, *p* = 0.017) but not CH samples (*p* = 0.40). All study predictors of the basic model taken together explained 13.03% of species‐level microbiome variation. Models using AD severity scores instead of AD presence were also non‐significant (EASI *p* = 0.93; SCORAD *p* = 0.93; v‐IGA AD *p* = 0.11).

**FIGURE 4:**
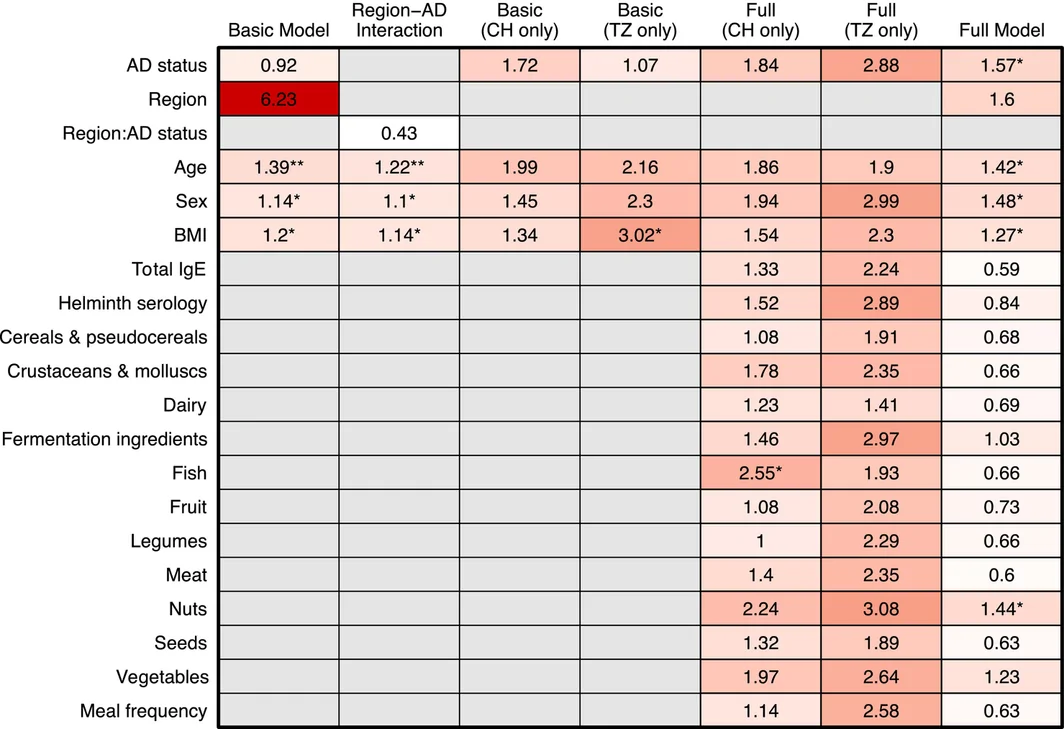
Proportions of variation in taxonomy (*R* ^2^ in %) explained by AD status, clinical covariables, standardized dietary intake, and circulating biomarkers quantified by PERMANOVA (Type III SS, 999 permutations stratified by region) based on species‐level Bray–Curtis dissimilarity. All statistical tests were two‐sided. *p < 0.05, **p < 0.01. AD, atopic dermatitis; BMI, body mass index; CH, Switzerland; IgE, Immunoglobulin E; TZ, Tanzania.

In a full model including AD status, age, sex, BMI, total IgE levels, major dietary categories, and the presence of intestinal parasites (type III SS; *n* = 118), 25.1% of species‐level variation could be explained by the model. Among study predictors, AD presence (*R* ^2^ = 1.57%, *p* = 0.012) and dietary intake of nuts (*R* ^2^ = 1.44%, *p* = 0.013) explained the largest amount of fecal microbiome structure (Figure 4), suggesting a modest but significant effect of AD on the microbiome when accounting for dietary factors.

### 3.3 Specific Microbial Genera and Species Are Associated With AD

To examine differentially abundant genera in AD vs. HC, we used an analysis model adjusting for region, age, and BMI. Bacteria of the genus CAG‐302 (NCBI: *Clostridium*) were less abundant in AD than in HC (log2FC = −0.023, *q* = 0.034). At the species level, *Faecalibacterium prausnitzii_D* (GTDB R226: *Faecalibacterium taiwanense*) was more abundant in AD (log2FC = 0.047, *p* = 0.004, *q* = 0.068), while CAG‐83 sp000435555 (GTDB R226: *Vescimonas* sp000435555, GCA_000435555.1) was more abundant in HC (log2FC = −0.038, *p* = 0.0032, *q* = 0.052, Figure 5, Supporting Information Table E4). There were no significantly differentially abundant genera or species for any covariate when subsetting the data by region.

**FIGURE 5:**
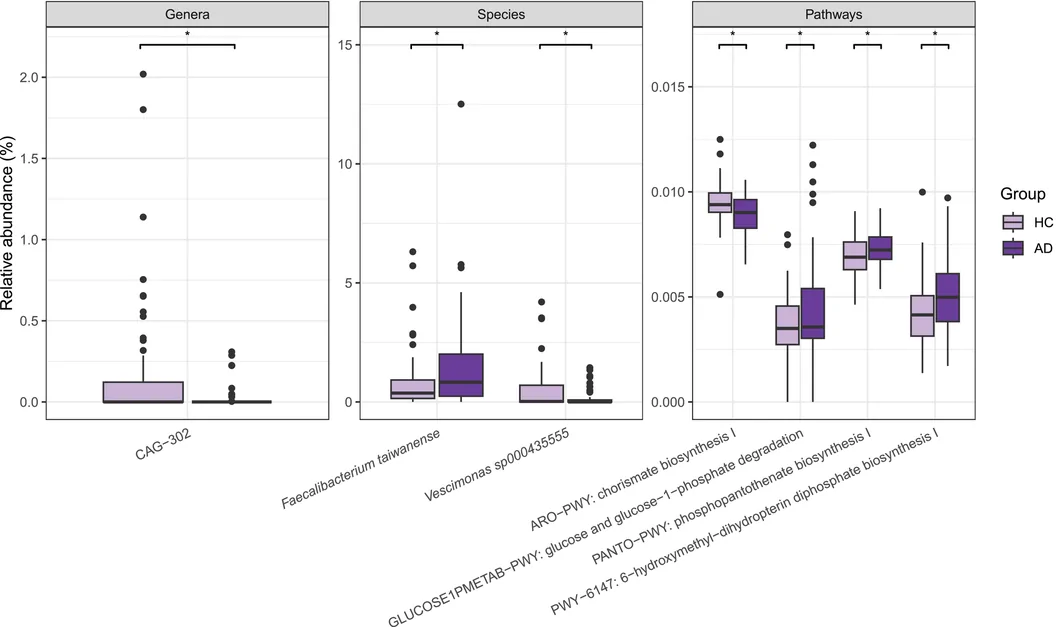
Boxplots of significantly differentially abundant genera, species, and MetaCyc pathways between AD and HC. *q < 0.1. AD, atopic dermatitis; HC, healthy control.

### 3.4 Metabolic Pathways Associated With AD

To get more functional insights, we mapped genes to metabolic pathways (MetaCyc) and analyzed potential differences between AD and HC. The analysis showed significant differences after controlling for age, sex, BMI, and region. Four MetaCyc pathways were differentially abundant: Chorismate biosynthesis I genes were less abundant in AD (log2FC = −0.0043, *p* = 0.0055, *q* = 0.056), while 6‐hydroxymethyl‐dihydropterin diphosphate biosynthesis I (log2FC = 0.0089, *p* = 0.0064, *q* = 0.062), phosphopantothenate (PPA) biosynthesis I (log2FC = 0.0043, *p* = 0.0067, *q* = 0.064), and glucose and glucose‐1‐phosphate degradation genes (log2FC = 0.014, *p* = 0.013, *q* = 0.099) were more abundant in AD (Figure 5, Supporting Information Table E4).

### 3.5 Atopic Comorbidities and Total IgE Levels

Certain allergic comorbidities have previously been associated with gut microbiome changes [14, 15]. In our cohort, AD patients with (*n* = 54) vs. without atopic comorbidities (*n* = 15) did not exhibit different microbiome compositions when adjusting for age, BMI, sex, and region (*p* = 0.86, Supporting Information Table E1).

We further wanted to explore a potential influence of total IgE levels on the gut microbiome. Total IgE levels were in the median 11.6 (95% CI 3.8–27.5) times higher in patients with AD and atopic comorbidities (ADAC) than in HC (Wilcoxon rank‐sum test: *p* < 0.001) and 20.1 (95% CI 4.0–107.9) times higher than in pure AD (Wilcoxon rank‐sum test: *p* < 0.001, Figure 6B). This pattern was also present when stratifying by region (Figure 6C). In our full PERMANOVA model across regions, total IgE levels (*R* ^2^ = 0.59%, *p* = 0.79) did not significantly influence the fecal microbiome composition (Figure 4).

**FIGURE 6:**
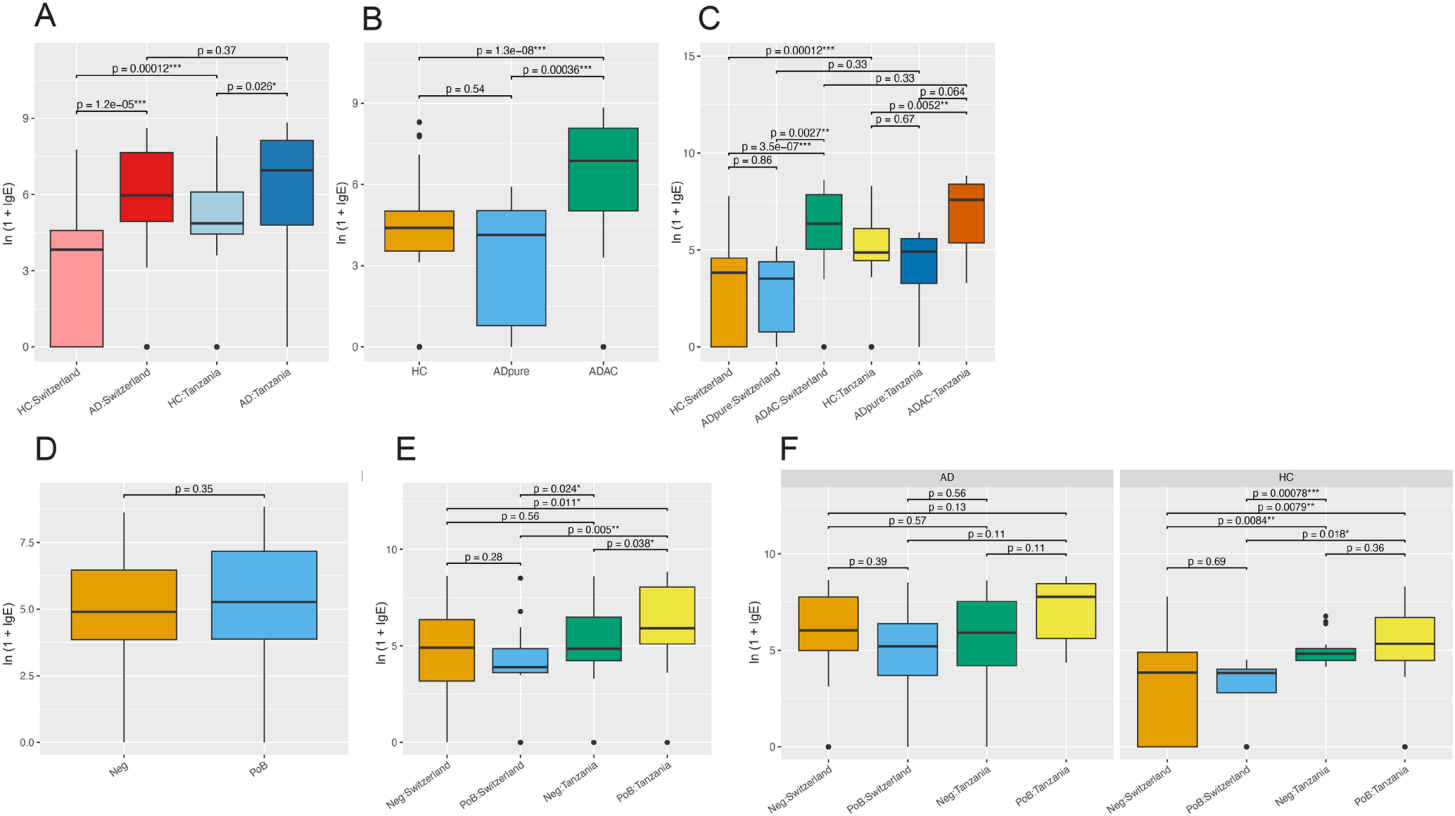
Boxplots evaluating differences in total IgE levels (natural logarithm ln(1 + IgE)) by (A) AD presence and region, (B) atopic comorbidity, and (C) atopic comorbidities and region using two‐sided Wilcoxon rank‐sum tests. (D) Boxplots evaluating differences in total IgE levels between individuals with Negative helminth screening (IgG ELISA) and one or more Positive or Borderline (PoB) helminth IgG screening. Tests were additionally stratified by (E) region (TZ vs. CH) or (F) region and AD status (AD vs. HC). *p < 0.05, **p < 0.01, ***p < 0.001. AD, atopic dermatitis; ADAC, atopic dermatitis with atopic comorbidities; ADpure, atopic dermatitis without atopic comorbidities; CH, Switzerland; HC, healthy control; IgE, Immunoglobulin E; IgG ELISA, immunoglobulin G Enzyme‐linked immunosorbent assay; TZ, Tanzania.

Interestingly, HC from TZ had significantly higher total IgE distributions than HC from CH (Wilcoxon rank‐sum test: *p* < 0.001, median TZ: 133 kU/l, median CH: 45 kU/l, Figure 6A). The estimated median ratio was 3.2, however with a wide 95% CI from 1.0 to 19.7.

Helminth infestation, which is often present in TZ, may underly this observation. A helminth serology screening showed that 27 TZ participants and 15 CH participants had Positive or Borderline (PoB) IgG results. TZ participants with PoB IgG results for any of the tested helminths showed significantly higher total IgE distributions than negative TZ individuals (Wilcoxon rank‐sum test: *p* = 0.038; estimated median ratio 1.7, 95% CI 0.13–7.2) and CH PoB individuals (Wilcoxon rank‐sum test: *p* = 0.005; estimated median ratio 6.5, 95% CI 1.0–61.8) (Figure 6E). Stratification by AD presence revealed that this effect was driven by HC participants, whereas the differences were not significant among AD participants (Figure 6F). Even in the absence of atopic diseases and parasite infestation among HC participants, TZ individuals showed higher total IgE level distributions than CH individuals (Wilcoxon rank‐sum test: *p* = 0.008; estimated median ratio 2.7, 95% CI 0.4–27.5). However, the wide confidence intervals of the median ratios, some of which included 1, indicate substantial uncertainty regarding the magnitude of the estimated effect. A positive parasite serology was, however, not a significant predictor of fecal microbiome composition in the full model (*R* ^2^ = 0.84%, *p* = 0.31; Figure 4).

### 3.6 Dietary Habits Differ Between Regions

We next tested our hypothesis whether and to which extent different geographical locations were associated with dietary differences assessed by our food questionnaire. Geographical location (CH vs. TZ) was associated with considerable differences in dietary habits. The following dietary categories were significantly less consumed in TZ compared to CH based on standardized scores: dairy products (*p* < 0.001), cereals and pseudocereals (*p* < 0.001), crustaceans and mollusks (*p* < 0.001), nuts (*p* < 0.001), fruit (*p* < 0.001), meat (*p* = 0.0071), and seeds (*p* < 0.001) (Supporting Information Figure E2). Meal frequency was lower in TZ (*p* < 0.001), with 93.65% of TZ participants eating 3 times per day or less compared to 64% of CH participants.

### 3.7 Differences in Microbiome Composition According to the Region and Associated Dietary Habits

Regarding the geographical location, there was no significant difference in alpha diversity between all participants from TZ vs. CH (*p* = 0.550, Figure 2B) or between AD patients from TZ vs. CH (*p* = 0.189, Figure 2C). Species‐level Bray–Curtis distances (*n* = 139) showed greater dispersion in TZ vs. CH samples (*p* = 0.001).

In a basic model, the region (TZ vs. CH) accounted for a substantial proportion of species‐level Bray–Curtis dissimilarity (*R* ^2^ = 6.23%), but the term was not statistically significant (*p* = 0.87; Figure 4). When subsetting the data by region, AD status was not a major driver of microbiome composition in CH (*p* = 0.13) or TZ (*p* = 0.89; Figure 4).

In our full model including AD status, age, sex, BMI, total IgE levels, major dietary categories, and the presence of intestinal parasites (type III SS; *n* = 118), not only the presence of AD (*R* ^2^ = 1.57%, *p* = 0.012), but also the consumption of nuts (*R* ^2^ = 1.44%, *p* = 0.013) accounted for the largest proportion of variation in fecal microbiome structure (Figure 4). Nut consumption was collinear with region, with standardized nut intake being higher in CH than TZ individuals (Mann–Whitney *U* test *p* < 0.001, Cliff's delta = 0.96; Supporting Information Figure E2). Additionally, variance partitioning revealed a larger shared contribution to microbial variation for region and nut intake (*R* ^2^ = 6.37%) relative to their unique effects (region *R* ^2^ = 0.96%, nuts *R* ^2^ = 1.28%). This suggests that their influence stems from overlapping/shared effects. Age (*R* ^2^ = 1.42%, *p* = 0.023), sex (*R* ^2^ = 1.48%, *p* = 0.014), and BMI (*R* ^2^ = 1.27%, *p* = 0.034) were also significant predictors of the fecal microbiome composition. On the other hand, region (*R* ^2^ = 1.6%, *p* = 0.79), total IgE levels (*R* ^2^ = 0.59%, *p* = 0.79), and the presence of a positive/borderline helminth serology (*R* ^2^ = 0.84%, *p* = 0.31) were not significant.

Overall, these findings suggest a modest but significant effect of nut intake and BMI on microbiome structure. Unlike age, sex, or the presence of AD, these factors are modifiable.

### 3.8 Bacterial Composition Differs Between Regions

On a genus level, we identified 54 significantly differentially abundant genera between regions in a linear Maaslin2 model accounting for age, sex, BMI, and AD presence (Figure 1C, Supporting Information Table E4). As region and dietary habits were largely collinear, dietary categories were not included in this model. *Prevotella* was significantly more abundant in Tanzanian participants (log2FC = 0.35, *p* < 0.001, *q* = 0.0078). *Alistipes* (log2FC = −0.17, *p* < 0.001, *q* < 0.001) and *Bacteroides* (log2FC = −0.15, *p* = 0.0033, *q* = 0.048) were significantly more abundant in Swiss participants.

When looking at the species level, 76 species were significantly differentially abundant in TZ vs. CH (Supporting Information Table E4). The most discriminative species for Tanzanian participants were *Prevotella copri_A* (NCBI: *Segatella copri* HF2112, GCF_024330295.1; log2FC = 0.13, *p* < 0.001, *q* = 0.0023) and *Prevotella* sp900313215 (NCBI: *Segatella copri* iAQ1173, GCF_009495355.1; log2FC = 0.12, *p* < 0.001, *q* = 0.0023). *Phocaeicola vulgatus* (log2FC = −0.19, *p* < 0.001, *q* = 0.0017) and *Alistipes putredinis* (log2FC = −0.13, *p* < 0.001, *q* < 0.001) were the most discriminative species for Swiss participants.

## 4 Discussion

The aim of our study was to decipher the influence of AD and different environmental factors such as geographical location and nutritional habits on the fecal microbiome composition.

We identified the AD status as the largest source of microbiome variation measured by Bray–Curtis beta diversity. On a species level, the abundance of *Faecalibacterium taiwanense* (GTDB R207: *Faecalibacterium prausnitzii_D*) was significantly higher, while the abundance of *Vescimonas sp000435555* was significantly lower in AD versus HC. On a genus level, CAG‐302 (NCBI: *Clostridium*) was less abundant in AD. AD severity and the presence of atopic comorbidities did not significantly contribute to the fecal microbiome variation.

Our hypothesis that there may be microbiome differences between CH and TZ was confirmed by our results, showing a higher abundance of *Prevotella* in TZ, whereas *Alistipes* and *Bacteroides* were significantly more abundant in CH. Geographical location was associated with distinct nutritional habits such as a lower standardized nut intake in TZ.

Our study showed similar levels of microbial alpha diversity between adult AD patients and HC. This is in line with several studies investigating children with AD [13, 49, 50]. Contrarily to our results, Ye et al. described a lower alpha diversity in AD than in HC; however, in a younger cohort of participants aged 6–22 years [51].

In our cohort, the presence of AD had a significant impact on the fecal microbiome variation (Bray–Curtis beta diversity). This is in line with Reddel et al. showing a significant difference in beta diversity between AD and HC in children [52]. Contrarily, a study in Korean infants did not find a difference in beta diversity between healthy children, transient AD, and persistent AD [53]. Two additional studies did also report no differences in beta diversity between AD and non‐AD children [13, 23]. Our results suggest a modest but potentially biologically significant effect of AD on the adult gut microbiome, which may not be present in children.

On a genus level, CAG‐302 (NCBI: *Clostridium*) was less abundant in AD patients. This is in line with other studies showing a significantly lower abundance of *Clostridium* in children/young adults with (persistent) AD [51, 53], but contrasts with findings from children aged 3 months [11]. Our findings were also interesting on a species level: *Faecalibacterium taiwanense* (GTDB R207: *Faecalibacterium prausnitzii_D*) was significantly elevated, and *Vescimonas sp000435555* significantly less abundant in our AD cohort compared to HC. To the best of our knowledge, we are the first to describe these changes in AD patients. *Faecalibacterium taiwanense* is an obligately anaerobic gram‐negative and butyrate‐producing bacterium, and it has only recently been classified as a novel species of the genus *Faecalibacterium* [54]. Regarding *Vescimonas sp000435555*, a recent study of Salazar et al. showed a negative correlation of *Vescimonas* (genus) abundance with BMI. The authors suggested a potential positive impact of *Vescimonas* due to its ability to metabolize carbohydrates and produce butyrate [55]. This could be particularly interesting, considering that butyrate (along with other short chain fatty acids) is discussed to play an anti‐inflammatory role by promoting regulatory T cell differentiation [23, 56, 57]. Enriching butyrate‐synthesizing bacteria may be a tempting therapeutic approach. In view of diverging results (lower abundance of *Vescimonas sp000435555*, but higher abundance of *Faecalibacterium taiwanense*), the exact role of butyrate producing bacteria in AD needs to be further elucidated. Also, we would like to highlight that associations do not imply causality.

Other treatment avenues exploiting the gut‐skin axis in AD may be fecal microbiota transplantation (FMT) from healthy donors [24]. This is under investigation, with promising results, such as a reduction in AD severity and decreased proportion of peripheral T helper 2 (Th2) cells [24, 25, 26, 27].

Interestingly, neither AD severity, nor the presence of atopic comorbidities in AD patients were associated with specific changes of the fecal microbiome. This is particularly intriguing considering previous studies showing microbial gut dysbiosis in patients with atopy, such as asthma or food allergy [15, 50, 58, 59]. As our HC participants were non‐atopic, we compared AD patients with vs. without atopic comorbidities, which might explain this discrepancy.

Intriguingly, we could show higher total IgE levels in TZ participants even in the absence of atopy and parasite infestation. Similar findings have been previously reported and may be due to genetic predispositions [60, 61, 62, 63]. Contrarily, a smaller study showed higher total IgE levels in CH AD compared to TZ AD [64]. Levin et al. could identify a single nucleotide polymorphism near HLA‐DQB1 as a significant predictor of total serum IgE levels in individuals of European and African descent and suggested that genetic variants may account for population specific differences in total IgE levels [62]. To our knowledge, however, no single genetic variant has been identified so far.

A second consideration relates to BMI, which was higher in the Tanzanian than in the Swiss cohort (*p* < 0.001; median difference 2.48 kg/m^2^; 95% CI: 1.33–3.78). Obesity has been linked to disturbed microbiome–immune interactions in inflammatory disease, as demonstrated by Michalovich et al. in asthma [14]. In our cohort, BMI did not differ significantly between AD patients and HC within either country (Table 1; CH median difference −0.20 kg/m^2^ (95% CI: −1.87 to 1.15, *p* = 0.74), TZ median difference 0.63 kg/m^2^ (95% CI: 1.70 to 2.62, *p* = 0.58)), and we therefore did not include it as a primary stratifying variable. To address this potential confounder, BMI was included as a covariate in our multivariable PERMANOVA model, where it emerged as an independent, albeit modest, predictor of microbiome composition (*R* ^2^ = 1.27%, *p* = 0.034); the previously reported between‐group differences nevertheless remained robust, supporting an effect of AD and dietary habits on the microbiome that is largely independent of BMI.

SCORAD and EASI scores were also notably higher in the Tanzanian cohort, and several factors are likely to contribute to this difference. First, the threshold for seeking medical attention may be higher for Tanzanian AD patients given more limited access to specialist healthcare, so that patients tend to present with more advanced disease. Second, more restricted availability of advanced systemic therapies (e.g., biologicals, Janus kinase inhibitors) in Tanzania may translate into less well‐controlled disease at the time of assessment. Additionally, both SCORAD and EASI rely partially on erythema, which is consistently underestimated on darkly pigmented skin, even by experienced clinicians [65]. Although our patients were assessed by two independent clinicians, the true disease severity in the Tanzanian cohort is therefore potentially even higher than the numerical scores suggest.

Understanding microbial metabolic pathways may provide additional clues to understand the gut‐skin axis in AD: In our study, phosphopantothenate (PPA) biosynthesis I‐related genes were more abundant in AD patients' fecal samples. PPA is an intermediate in the coenzyme A synthesis pathway, which in turn is involved in many metabolic and biosynthetic pathways [66, 67]. PPA is formed by phosphorylation of the essential vitamin B5 (pantothenate) which is obtained through dietary uptake in humans. Vitamin B5 is synthesized by bacteria, plants, and fungi [68]. Only one study has linked coenzyme A and AD so far: Higashi et al. hypothesize that breastmilk‐derived coenzyme A could lead to a higher Th2 adjuvant activity and thereby promote AD in toddlers [69]. It remains to be elucidated whether and by which mechanisms coenzyme A may have similar effects in the gut.

Additionally, different pathways involved in bacterial folate production were dysregulated in AD vs. HC. First, chorismate biosynthesis genes were less abundant in AD. Chorismate is a precursor for folate biosynthesis and for certain amino acids (tryptophan, tyrosine, and phenylalanine) [70, 71]. Chorismate biosynthesis is dependent on glycolysis [70], however, there was a higher abundance of glucose and glucose‐1‐phosphate degrading genes in our AD cohort. Contrarily, Ta et al. have described an accumulation of pre‐glycolysis metabolites such as D‐glucose, D‐galactose, lactose, and lactate in allergen‐sensitized infants with AD [72]. On the other hand, the 6‐hydroxymethyl‐dihydropterin diphosphate biosynthesis I pathway genes were more abundant in AD in our cohort. It is part of de novo folate biosynthesis in bacteria [73, 74]. An upregulation of folate synthesis has been shown in the exponential growth phase of bacteria [71]. As humans cannot synthesize folate, the two possible sources are (I) through oral/dietary intake of folates and (II) through their fecal microbiome [75]. A deficiency of folic acid has been linked to disruption of intestinal integrity, and a protective effect of folate on autoimmune diseases has been proposed in the literature (reviewed in [74]). The intake of folic acid during pregnancy is recommended to prevent neural tube defects [76]. Yet, multiple studies discuss a potential harmful effect of high maternal folate levels on childhood AD. A study conducted in 2012 suggested a positive association between high maternal serum folate levels and the development of AD [77]. Similarly, a prospective study by Ye et al. stated an increased risk of AD with higher maternal red blood cell folate levels during early gestation [78]. However, in both studies, there was no association between the intake of folic acid supplements and the child's risk of AD [77, 78]. Additionally, Roy et al. did not find an association between high maternal folate levels and childhood atopic dermatitis [79]. Even if meta‐analyses suggest that there is no association between folic acid supplementation during pregnancy and the development of AD in childhood [80, 81], our results of dysregulated pathways involved in bacterial de novo folate biosynthesis in the intestine of AD patients raise the question of whether the folate metabolism of intestinal bacteria could play a role in the pathogenesis of AD.

Regarding the question as to regional differences and its possible reasons, we hypothesized that distinct dietary habits may play an important role. Our results supported the idea that regional differences are considerably driven by nutrition as an important environmental exposure.

Our study detected a significant association between standardized nut consumption and the fecal microbial composition. Standardized nut consumption was higher in CH than in TZ individuals. Additionally, the meal frequency was significantly lower in TZ than in CH. However, as this was an observational study, dietary data relied on self‐reported food intake rather than standardized diets, which may have introduced reporting bias. Furthermore, we identified a higher abundance of *Prevotella* in TZ, whereas *Bacteroides* and *Alistipes* were more abundant in CH. This confirms data that regional differences and associated nutritional habits can significantly influence the fecal microbiome [29, 30]. A more plant‐ and fiber‐based diet has been associated with enrichment of fiber‐degrading taxa such as *Prevotella*, whereas frequent intake of protein and fat is typically associated with *Bacteroides*‐dominated communities [29, 30].

In line with our results, Wu et al. have described two distinguished enterotypes depending on dietary patterns in a healthy population: While a protein and animal fat rich diet was associated with the *Bacteroides* enterotype and an additional presence of *Alistipes*, a more carbohydrate‐based diet was associated with the *Prevotella* enterotype [31]. Even if the microbiome composition started to change a day after introducing diet changes, the enterotypes remained stable [31]. Both *Prevotella* and *Bacteroides* genera belong to the phylum of Bacteroidota [82]. As *Bacteroides* are bile‐tolerant bacteria, their higher abundance in an animal‐based diet makes sense from a biological point of view [29]. On the other hand, *Prevotella* is able to convert plant polysaccharides and fibers into short chain fatty acids such as butyrate, acetate, or propionate [82]. Truter et al. analyzed the gut microbiome of the Namibian Ju/'Hoansi and detected a high abundance of *Prevotella*. Interestingly, *Alistipes* was primarily detected in villages with higher antibiotic use [82]. Schnorr et al. investigated the gut microbiome of Tanzanian Hadza and detected an enrichment of *Prevotella* and a lower relative abundance of *Bacteroides* compared to an Italian cohort [83]. Another study could show a loss of *Prevotella* strains and fiber‐degrading enzymes in immigrants from Thailand to the United States, whereas *Bacteroides* strains expanded [30]. Their definition of a “non‐western” *Prevotella* dominant microbiome and a “western” *Bacteroides* dominant microbiome is in line with our findings with a higher relative abundance of *Prevotella* in TZ and a higher relative abundance of *Bacteroides* in the CH cohort. Reddel et al. additionally described higher levels of the genus *Bacteroides* in AD [52], which could not be confirmed by our study.

What is currently missing is longitudinal data, which would help to clarify whether and how microbiome and dietary changes affect the course of AD. This may ultimately help in providing more tailored dietary recommendations to our patients. In addition, a concurrent assessment of the metabolome, in combination with mechanistic studies, could provide new pathomechanistic insights into the interplay between gut microbiome, diet, and AD‐associated inflammation.

### 4.1 Strengths and Limitations

The strengths of this study lie in the unique cohort, including patients from both Europe and Africa, its prospective design and the comprehensive approach including sequencing data, clinical data, as well as information about nutritional habits. Additionally, we focus on an adult population, whereas prior studies of the fecal microbiome in AD mostly included children. As a limitation, it should be mentioned that stool sampling was performed cross‐sectionally and not longitudinally to assess potential changes over time. Dietary habits were self‐reported using a newly developed semi‐quantitative food‐frequency questionnaire to capture food intake habits and were not assessed at the nutrient level. These results should therefore be interpreted with caution. Lastly, as functional pathways were inferred computationally rather than directly measured, experimental validation in future studies is needed. Also, we would like to emphasize that the described associations do not imply causality.

## 5 Conclusions

AD patients in our study exhibited fecal microbial dysbiosis. Geographical location (CH vs. TZ) was associated with microbiome changes and may be due to different nutritional habits. Our findings support the “gut‐skin axis” concept and strengthen the rationale for therapeutic approaches modulating the gut microbiome in AD.

## Author Contributions

Study conceptualization and design: D.F., M.‐C.B. Funding acquisition: D.F., M.‐C.B. Recruitment of patients and/or acquisition of data: D.F., G.M., N.M. Processing of biomaterial: D.F., N.L., A.W., G.S., F.G. Statistical computation: N.F. Drafting of the manuscript: D.F., N.F. Critical revision of the manuscript for important intellectual content: D.F., N.F., N.L., A.W., M.D., C.L., G.M., N.M., G.S., M.S., F.G., D.M., J.E.M., P.S.‐G., M.‐C.B.

## Funding

This work was supported by LEO Fondet (LF‐OC‐20‐000418), Vontobel‐Stiftung (0456/2024), Bruno Bloch Stiftung.

## Ethics Statement

This study has been approved by the cantonal ethics committee of Zurich, Switzerland (BASEC Nr. 2021–01869) and the National Institute for Medical Research in Tanzania (NIMR Nr. 3708).

## Consent

All patients gave their written informed consent to participate in this study.

## Conflicts of Interest

Marie‐Charlotte Brüggen reports a grant from the LEO foundation for this project. Further grants from LEO foundation, LEO Pharma, Swiss National Science Foundation, Innovation Pool, GSK, Astra Zeneca; speaker and/or consulting fees from Sanofi, Almirall, GSK, LEO Pharma, Abbvie are not related to the current project. Danielle Fehr reports grants from the Vontobel Stiftung and from the Bruno Bloch Stiftung that were used for this project. Claudia Lang is or was a speaker, advisor, or investigator for AbbVie, EliLilly, LEO, Novartis, Pfizer, and SanofiGenzyme. Peter Schmid‐Grendelmeier was speaker, advisor, or investigator for AbbVie, Astra Zeneca, Biomed, EliLilly, Galderma, Glaxo Smith Kline, LEO, L'Oreal, Novartis, Pfizer, Pierre Fabre, and SanofiGenzyme. The rest of the authors have no conflicts of interest to declare.

## Supporting information


**Figure E1.** Phylogenetic tree illustrating the microbial population structure across samples. Text around the plot labels the top 6 most abundant bacterial families. The most abundant genera are labeled with a capital letter on the plot and defined in the top left corner. Colors indicate the most abundant phyla and are labeled in the top right corner.

**Figure E2.** Boxplots of standardized dietary intake scores by region (CH *n* = 72; TZ *n* = 46) collected by questionnaire. Standardized scores are z‐scores of the median dietary intake score across collapsed subcategories. Dietary subcategories used to create each of the reported major categories can be found in the supplements (Supporting Information Table E2). *p*‐Values were calculated using Mann–Whitney *U* tests and adjusted for multiple hypothesis testing using the Benjamini‐Hochberg procedure.

**Table E1.** PERMANOVA model output for proportions of variation in taxonomy (*R* ^2^) explained by AD status, covariables, standardized dietary intake, and circulating biomarkers quantified by PERMANOVA (Type III SS, 999 permutations stratified by region) based on species‐level Bray–Curtis dissimilarity.

**Table E2.** Dietary subcategories that were used to create each of the reported major dietary categories.

**Table E3.** Table of alpha diversity values (Shannon index) for all samples and calculated medians for group comparisons of interest.

**Table E4.** Significant Maaslin2 results for differentially abundant species, genera, and MetaCyc pathways.

**Data S1:** Supplementary Methods

## Data Availability

The datasets generated and analyzed during the current study are available from the corresponding author upon reasonable request. The shotgun metagenomics data of stool samples that support the findings of this study are openly available in the European Nucleotide Archive at https://www.ebi.ac.uk/ena/browser/home, reference number PRJEB101120.
