# Supplementary material for: Dietary Habits and Atopic Dermatitis Significantly Influence the Fecal Microbiome
Source: Allergy. 2026 Jul 28;81(10):3584–98. doi: 10.1111/all.70459 (PMC13646880; doi:10.1111/all.70459)
Supplement: Supplementary file 1 [file ALL-81-3584-s004.pdf]

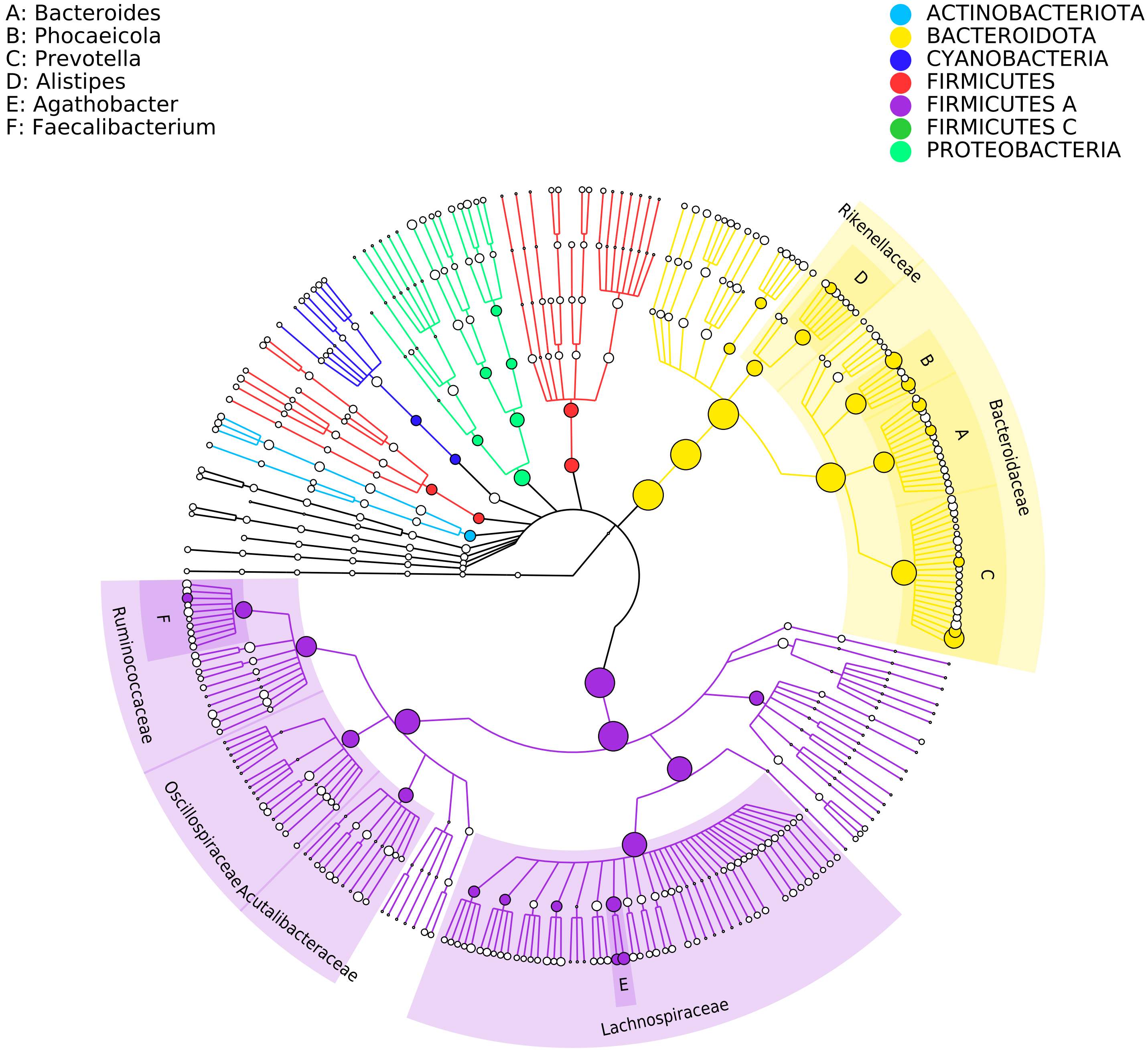

A: Bacteroides
B: Phocaeicola
C: Prevotella
D: Alistipes
E: Agathobacter
F: Faecalibacterium
ACTINOBACTERIOTA
BACTEROIDOTA
CYANOBACTERIA
FIRMICUTES
FIRMICUTES A
FIRMICUTES C
PROTEOBACTERIA
Rikenellaceae
Bacteroidaceae
Ruminococcaceae
Oscillospiraceae
Acutalibacteraceae
Lachnospiraceae
A
B
C
D
E
F
