# Supplementary material for: Dietary Habits and Atopic Dermatitis Significantly Influence the Fecal Microbiome
Source: Allergy. 2026 Jul 28;81(10):3584–98. doi: 10.1111/all.70459 (PMC13646880; doi:10.1111/all.70459)
Supplement: Supplementary file 2 [file ALL-81-3584-s005.pdf]

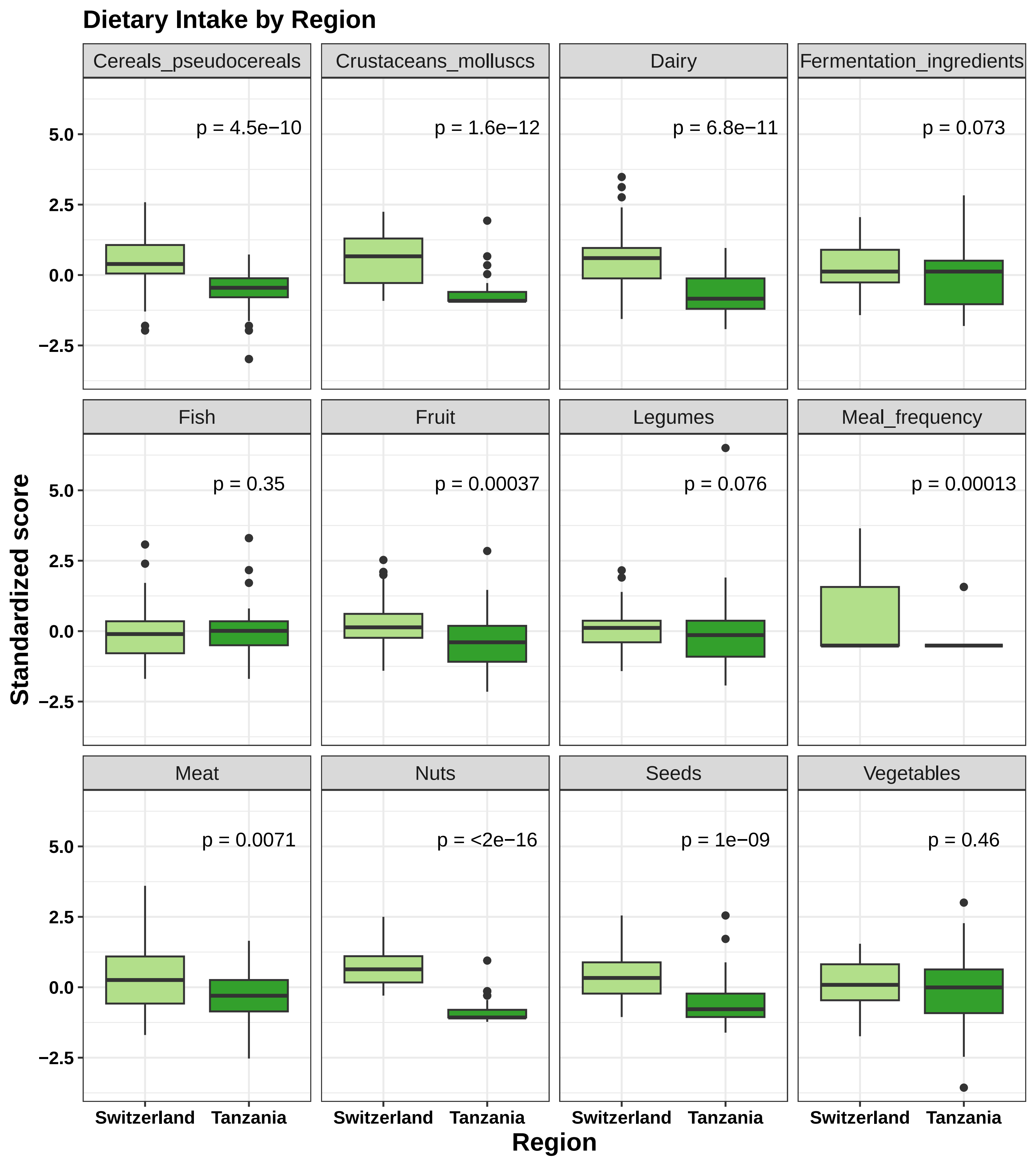

Dietary Intake by Region
Cereals_pseudocereals
p = 4.5e−10
Crustaceans_molluscs
p = 1.6e−12
Dairy
p = 6.8e−11
Fermentation_ingredients
p = 0.073
Fish
p = 0.35
Fruit
p = 0.00037
Legumes
p = 0.076
Meal_frequency
p = 0.00013
Meat
p = 0.0071
Nuts
p = <2e−16
Seeds
p = 1e−09
Vegetables
p = 0.46
Standardized score
5.0
2.5
0.0
−2.5
Switzerland
Tanzania
Region
