# Supplementary material for: Dietary Habits and Atopic Dermatitis Significantly Influence the Fecal Microbiome
Source: Allergy. 2026 Jul 28;81(10):3584–98. doi: 10.1111/all.70459 (PMC13646880; doi:10.1111/all.70459)
Supplement: Supplementary file 3 [file ALL-81-3584-s007.pdf]

Supplementary Methods

Dietary Habits and Atopic Dermatitis Significantly Influence the Fecal Microbiome

Eligibility criteria

AD patients were included in the study if they were 18 years or older and suffering from active AD at the time of inclusion. Exclusion criteria were other active skin diseases, known active inflammatory diseases other than AD/atopic conditions. HC were included if they were 18 years or older. Exclusion criteria were any active skin disease, known atopic diseases and known active inflammatory diseases. The following criteria were enforced as exclusion criteria for both AD and HC participants: known or suspected immunosuppressive disease; any immunosuppressive or immunomodulatory treatment; any signs of active infection; any systemic antibiotic, antimycotic, antiviral treatment, or phototherapy within 4 weeks before inclusion; any active neoplasia; any surgery, infarction, embolism, or thrombosis in the last 2 months; or if they were not able to follow the study procedures. Out of a total of 160 participants, stool samples were available of 140 participants who were therefore included in the present project.

Statistical analysis

Preprocessing

The FASTQ files were quality-checked with fastp version 0.23.4 and MultiQC version 1.14, removing any remaining adapter sequences, reads shorter than 100 bp, and reads with an average Phred quality score below 20. Human reads were removed by

mapping reads to the human reference genome (hg38) with Bowtie2 version 2.5.1 and retaining unmapped read pairs for downstream analysis.

Taxonomic classification

Genus-level and species-level relative abundances for each sample were calculated with MetaPhlAn4 v4.1.1 using the ChocoPhlAn marker database "mpa_vJun23_CHOCOPhlAnSGB_202403". To estimate the mean proportion of unclassified sequences across samples, a second run of MetaPhlAn4 with the "--unclassified_estimation" flag was performed. Downstream analyses utilised the program's default output (i.e. unclassified proportion ignored in the relative abundance calculations). The utility scripts provided with MetaPhlAn4 were used to merge taxonomic profiles across samples and convert National Center for Biotechnology Information (NCBI)-based species-level genome bin (SGB) taxonomic identifiers to Gene Taxonomy Database (GTDB) identifiers. The merged abundance table for all samples was used to produce abundance heatmaps at the genus- and species-level. Additionally, a cladogram containing all of the study's samples together was generated with GraphPhlAn v1.1.3. The SGB-GTDB conversion table provided with MetaPhlAn4 v4.4.1 was built with GTDB Release 207 (R207; April 2022); taxa are referenced using R207 identifiers or identifiers from the most recent GTDB Release 226 (R226; April 2025) if a change has occurred between versions. NCBI identifiers are also referenced with the source taxonomy noted for clarity.

Microbial composition and discriminative taxa

Alpha diversity values were calculated using utility scripts provided with MetaPhlAn4. Shannon diversity indices were calculated at the species level and compared between the four AD status/region groups (AD-Switzerland, HC-Switzerland, AD-

Tanzania, HC-Tanzania) with pairwise Wilcoxon rank sum tests. Bray-Curtis distances were calculated from species-level relative abundance values, excluding taxa present in < 10% of samples or with < 0.01% mean relative abundance, using the R package vegan v2.6-4. Associations between Bray-Curtis distances and AD status (presence, severity), atopic comorbidities, dietary intake, and further variables (e.g., total immunoglobulin E (IgE), parasites) were tested using PERMANOVA (vegan::adonis2) with type III sum of squares (SS; by = "margin"), allowing assessment of each predictor's unique contribution. All models included age, sex, and BMI as covariates, and predictors with $p < 0.05$ were considered significant. Homogeneity of group dispersions was assessed with PERMDISP (vegan::betadisper). For cross-regional models, permutations were stratified by region (CH vs. TZ) to account for greater dispersion in TZ samples ($p = 0.001$). Variance partitioning of Bray-Curtis dissimilarities was performed using vegan::varpart. Differences in standardized diet scores between groups were tested using Mann–Whitney U tests; p-values were adjusted for multiple testing using the Benjamini-Hochberg procedure and effect sizes were calculated using Cliff's delta where appropriate (R package rcompanion v2.4.36).

The abundance of genes associated with microbial metabolic pathways (MetaCyc) was evaluated with HUMAnN v3.9. Differentially abundant species, genera, and pathways between AD cases and controls were evaluated with MaAsLin2 v1.12.0. Linear models were used (analysis_method = "LM") with all covariates treated as fixed effects. A basic model adjusting for age, sex, BMI, and region was prioritized to reduce variance inflation from high collinearity between region and dietary intake categories. Feature abundances were the arcsine square root-transformed relative abundances (transform = "AST") of species, genera, and MetaCyc pathways,

respectively. Inputs were filtered with a minimum relative abundance of 0.1% and minimum prevalence of 20%. Multiple tests were controlled with FDR correction calculated by the Benjamini-Hochberg method with q-values < 0.1 (MaAsLin2 default = 0.25) being deemed significant. References for computational packages can be found in the main manuscript.
